# The symmetry-induced numerosity illusion depends on visual attention

**DOI:** 10.1038/s41598-023-39581-w

**Published:** 2023-08-02

**Authors:** Paula A. Maldonado Moscoso, Giuseppe Maduli, Giovanni Anobile, Roberto Arrighi, Elisa Castaldi

**Affiliations:** 1 Department of Neuroscience, Psychology, Pharmacology and Child Health, University of Florence, Florence, Italy; 2 Center for Mind/Brain Sciences (CIMeC), University of Trento, Rovereto, Italy

**Keywords:** Human behaviour, Attention, Perception

## Abstract

Symmetry is an important and strong cue we rely on to organize the visual world. Although it is at the basis of objects segmentation in a visual scene, it can sometimes bias our perception. When asked to discriminate numerical quantities between symmetric and asymmetric arrays, individuals tend to underestimate the number of items in the symmetric stimuli. The reason for this underestimation is currently unknown. In this study we investigated whether the symmetry-induced numerosity underestimation depends on perceptual grouping mechanisms by depriving attentional resources. Twenty-six adults judged the numerosity of dot arrays arranged symmetrically or randomly, while ignoring a visual distractor (single task) or while simultaneously judging its color and orientation (dual-task). Diverting attention to the concurrent color–orientation conjunction task halved the symmetry-induced numerosity underestimation. Taken together these results showed that the bias in numerosity perception of symmetric arrays depends—at least partially—on attentional resources and suggested that it might originate from the recruitment of attentional dependent incremental grouping mechanisms.

## Introduction

One of the biggest challenges the visual system needs to take on to reconstruct an accurate and coherent representation of the external world is to identify meaningful objects within the visual image and segregate them from the background. To achieve these goals features defining individual objects’ borders must be identified and grouped together. Gestalt psychologists described several strategies the visual system might exploit to group and organize the visual scene, including objects’ continuity, proximity, similarity, common region, common fate, connectedness and symmetry^1–3^. Object segregation is crucial to form a gist of the number of objects in a scene, that is for numerosity perception, an ability present across the animal kingdom, from insects to humans^4–7^. Numerosity perception is mediated by the subitizing system, which supports fast and errorless numerical perception of up to 4 objects^8,9^, and by the approximate number system (ANS), a slower and less precise process that is recruited for the perception of larger numerosities, provided that objects in the ensemble are sparse enough to be segregated from one another. When objects are too cluttered to be individually identified, a third system that processes texture density is activated^10–12^.

Interestingly, Gestalt cues can bias the perception of numerosity in both human and non-human animals. For example, the numerosity of items regularly arranged in space tends to be overestimated, compared to irregular patterns (e.g., Regular-Random numerosity illusion, coherence illusion and solitaire illusion)^13–19^, whereas clustering (or grouping) by proximity, color, motion, similarity and symmetry elicits underestimation of perceived numerosity^20–24^.

One of the strongest and probably the most investigated numerosity illusion elicited by grouping cues is that induced by connecting items with thin lines (connectedness illusion)^25–29^, or by enclosing them in ovals^30^. In this illusion, connected items are strongly underestimated with the extent of underestimation monotonically increasing with the number of paired items, even when the connection is illusory as that mediated by Pacman-like stimuli^23,31,32^. This phenomenon has been taken as evidence that numerosity mechanisms operate on segmented objects rather than on individual local elements. Supporting this idea, the underestimation effect resulting from the connectedness illusion was stronger for moderate numerosity, processed by the ANS system (estimation range) and greatly reduced for cluttered stimuli, processed by the texture-density system^27^, suggesting that numerosity, but much less texture perception, requires object’s segmentation. Despite robust, the magnitude of the connectedness-induced underestimation effect varies between individuals. A significant portion of interindividual variation in the strength of this perceptual illusion is captured by individuals’ perceptual style, as indexed by a self-reported Autistic Quotient questionnaire (AQ) with lower effect in individuals with higher autistic traits and more local perceptual style, compared to those with lower autistic traits and a more global perceptual style^33^. Other factors such as visual attention can also modulate the connectedness illusion strength. When attention is divided between the numerosity task and a distractor task, the illusion is considerably reduced^34^ in line with the idea that attentional resources are implicated in the grouping processes^35–39^; for a review see^40^.

Another Gestalt cue strongly affecting numerosity perception is symmetry. Symmetry is of special interest in the visual domain, manly for its biological relevance^41–43^ prompting figure-ground segregation^44–48^. As for the connectedness illusion, symmetric dot patterns are usually perceived as less numerous compared to random patterns^22,49^. Why symmetric patterns lead to numerosity underestimation is currently unknown. According to the hypothesis advanced by Apthorp and Bell^22^, the underestimation occurs because symmetric arrays contain redundant information and participants might limit their attention to one-half of the display, resulting in underestimation the number of perceived elements^22^. Another, still unexplored, explanation is based on grouping. Similarly to the connectedness/enclosed illusions, our visual system might tend to perceptually connect symmetrical (physically unconnected) elements to form coherent shapes^45–47,50^, resulting in numerosity underestimation. In line with this hypothesis a previous study traced several parallelisms between the numerosity underestimation effect elicited by connectedness and symmetry cues^49^. Similarly to the numerosity underestimation induced by connectedness, the effect induced by symmetry is stronger for moderate numerosities compared to high numerosities (in the texture-density range), suggesting that the illusion might pick on the ANS system while it attempts to operate on segmented objects, to extract the overall ensemble numerosity. Furthermore, similarly to what observed for the connectedness-illusion, the strength of numerosity underestimation induced by symmetry seems also to depend on individuals’ perceptual style, being weaker for individuals with relatively higher autistic traits^49^.

In the current study, we aimed to further investigate the origin of the symmetry-induced numerosity underestimation effect by examining its dependence on attentional resources. Specifically, we tested whether the numerosity underestimation induced by symmetrical stimuli is modulated by the amount of visual attention resources available. To this aim, participants were engaged in a discrimination task comparing the relative numerosity of symmetric and random dot patterns. Attentional resources were manipulated with a dual-task procedure so that the numerosity task was performed while performing or not a concurrent, central color-orientation distractor task. If the numerosity underestimation elicited by symmetry depends on grouping mechanisms we expect it to be reduced in the dual-task condition, as previously observed when grouping is triggered by connectedness^34^.

## Methods

### Participants

To estimate the required sample size, we performed a power analysis (for a t-test between the mean underestimation effect of random and symmetric stimuli) extracting the effect size from a similar previous study investigating the effect of symmetry on numerosity perception^49^. With an effect size of 0.75, an *α* = 0.05 and a required power of 0.95, the analysis suggested a sample of 26 participants.

A total of twenty-six young adults (15 females; mean age = 24.57, std = 2.3, range = 19–30 y.o.) participated in the study. Participants were all university students with normal or corrected-to-normal vision. The experimental procedures were approved by the local ethics committee (Commissione per l’Etica della Ricerca, University of Florence, July 7, 2020, n. 111). The research was performed in accordance with the Declaration of Helsinki and informed consent was obtained from all participants before the experiment.

### Apparatus and stimuli

Stimuli were generated and presented with PsychToolbox routines for Matlab (ver. R2016b, The Mathworks, Inc.). Stimuli were presented on an iMac 27″ monitor subtending 42° × 24° at a viewing distance of 57 cm (screen resolution: 2560 × 1440; refresh rate: 60 Hz). The experiment was performed in a quiet and dimly light room.

**Figure 1:**
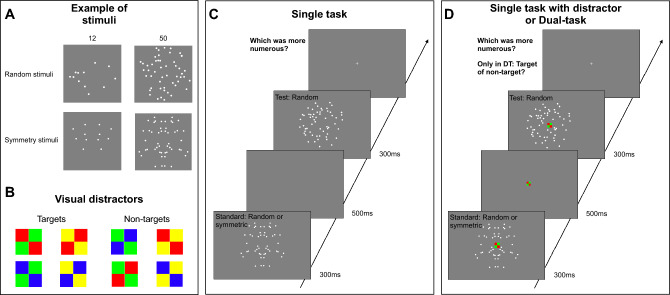
(**A**) Example of stimuli for random and symmetric dot arrays (**B**). Visual distractors used in the single task with distractor (ST-WD) and in the dual-task (DT) conditions. (**C**,**D**) Timeline of the single task (**C**) and single task with distractor/dual-task (**D**) conditions.

Stimuli were arrays of white dots (0.3° diameter), which were distributed either randomly or symmetrically around the vertical axis. Dots were located at least 0.3° from each other and constrained to fall within a virtual circle of 10° diameter. Dots possible locations did not cover a central area (1.6° × 1.6°) to avoid overlap with the location of the distractor stimuli (see below). Symmetric displays were created by first randomly drawing dots in half of the array space and then flipping their coordinates around the vertical axis (Fig. 1A)^49^. The average convex hull of the symmetric and random stimuli was comparable (mean = 45 deg^2^, std = 14 and mean = 45 deg^2^, std = 15, for symmetry and random condition respectively, t(11,014) = 3.9, p > 0.99).

On each trial, two sequential arrays of dots were centrally presented: one symmetrical and the other asymmetrical (symmetry condition), or both asymmetrical (random condition). Stimuli were briefly (300 ms) presented with an inter-stimulus interval of 500 ms (Fig. 1C). The standard numerosity (8, 12, 24, 50 dots in different sessions) was randomly assigned as the first or the second stimulus. The numerosity of the test stimulus changed following an adaptive staircase QUEST algorithm^51^.

Participants were tested with three conditions: two single task and one dual-task conditions. The two single task conditions were designed to test numerosity perception of symmetric arrays at baseline (no visual distractor) and while a visual distractor, that had to be ignored, was simultaneously presented onscreen. The latter condition aimed to test whether a mere sensory load was sufficient to change the effect of symmetry on numerosity perception. In both single task conditions, i.e. single task without distractors (ST) and single task with distractor (ST-WD), participants indicated which array contained more dots by pressing the left or the right arrow to select the first or the second stimulus respectively. In the dual-task condition (DT) participants performed the same numerosity discrimination task, while their visuo-spatial attentional resources were also deployed on a concurrent color–orientation conjunction task. The visual distractor (Fig. 1B) comprised a central square (1° × 1°) including four squares each colored either green, yellow, blue and red. Depending on the conjunction of colors and spatial arrangement, the visual distractor was either a target or not^52^. Specifically, four (among eight possible) color arrangements identified the target (two green squares along the right diagonal, or two yellow squares along the left diagonal; Fig. 1B). Participants were asked first to indicate whether the presented stimulus was a target or not by pressing the “t” or the “y” key respectively and then to perform the numerosity discrimination task (Fig. 1D).

For each standard numerosity, participants performed two sessions of 50 trials for a total of 800 trials (400 for symmetry and 400 for random condition) for each task (ST, ST-WD and DT).

### Data analysis

Statistical analyses were performed using Jasp (version 0.16, The Jasp Team 2021) and Matlab (version R2016b, The Mathworks, Inc., http://mathworks.com, 15 September 2016).

For each standard numerosity (8, 12, 24, 50) the proportion of trials for which the test stimulus appeared to be more numerous than the standard was plotted as a function of the numerosity of the test and fitted with a cumulative Gaussian error function (psychometric function). The point of subjective equality (PSE) was defined as the physical numerosity of the test yielding 50% of "test more numerous". To obtain a measure for the underestimation effect, we calculated a bias index for each participant, condition and standard numerosity, which was averaged across participants and quantified as follows:

1 $$Bias=\left(\frac{{PSE}_{N}}{N}-1\right)\times 100$$

where PSE_N_ is the PSE for standard numerosity N.

The just notable difference (JND) was calculated as the difference in numerosities between the 50% and 75% points on the psychometric function. To get a single measure for discrimination thresholds, we normalized the JND by each standard numerosity (N) obtaining a dimensionless psychophysical index (Weber fraction; Wf):

2 $$Wf= \frac{JND}{N}$$

Data were analyzed by repeated measures ANOVAs and post-hoc t-tests. Effect sizes were reported as η^2^ or Cohen’s d, and p-values were corrected for multiple comparisons with the Bonferroni method and reported as p_bonf_. Performance to the color–orientation conjunction task was analyzed as the proportion of correct responses when the numerical stimuli were symmetric and when they were random. To compare the performance between the two conditions we performed a non-parametric Wilcoxon signed-rank test (W).

## Results

### The effect of symmetry on numerosity perception

**Figure 2:**
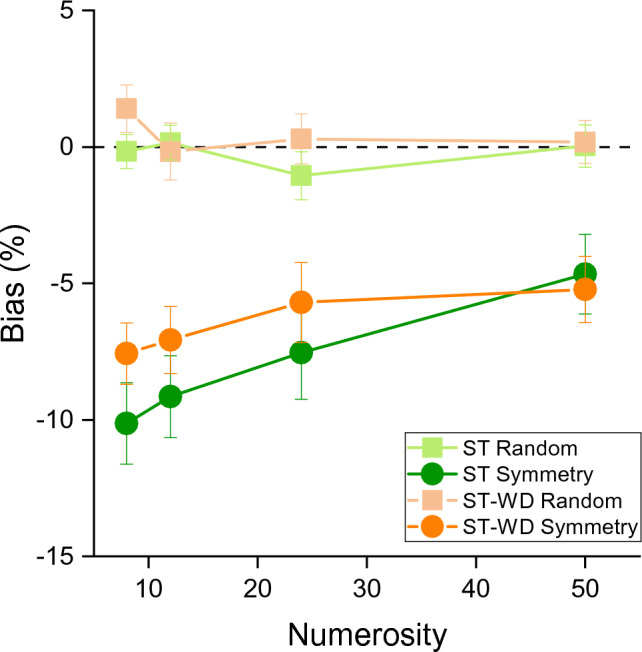
Average Bias as a function of the standard numerosities, for the two conditions (symmetry and random) in single task (ST) and single task with distractor (ST-WD). Symbols represent average across participants. Error bars represent ± 1 s.e.m.

At first, we assessed the numerosity underestimation illusion induced by symmetric arrays and tested whether the effect was modulated by the mere presence of a to-be-ignored distractor (passive sensory load). Figure 2 shows the PSE biases as a function of numerosity with negative values indicating underestimation of the symmetric stimuli, compared to random arrays. By visual inspection it is evident that the results were independent by the presence or absence of the to-be-ignored distractor. In the random condition, whether the distractor was present or not, the biases were all around zero, as expected, indicating an overall accurate performance (single task without distractor: mean = − 0.25, std = 1.8; single task with distractor: mean = 0.42, std = 2.3). In the condition in which the elements were arranged symmetrically a reliably underestimation of around 7% emerged, again independently by the presence of the distractor stimulus (single task without distractor: mean = − 7.87, std = 5.9; single task with distractor: mean = − 6.4, std = 5.3).

To statistically test these differences, we entered the biases in a RM ANOVA with numerosity (4 levels: 8, 12, 24, 50), condition (2 levels: symmetry and random) and single task type (with and without visual distractor) as within-subjects factors. The significant main effect of condition (F(1,25) = 63.72, p < 0.001, η^2^ = 0.34), suggested that the numerosity of symmetric stimuli was underestimated compared to the numerosity of random stimuli. Interestingly, the lack of significant interaction between single task type and condition (F(1,25) = 0.64, p = 0.43, η^2^ = 0.001) suggested that the amount of symmetry-driven underestimation was comparable across single task types, and therefore that the mere presence of the ignored visual distractor did not alter numerosity perception of symmetric vs random arrays. The interaction between numerosity and condition was significant (F(3,75) = 4.05, p = 0.01, η^2^ = 0.019) indicating a progressively smaller, albeit always significant, underestimation of symmetric stimuli compared to random arrays with increasing numerosity (t(25) for symmetric vs random for all numerosities > 4.08, p_bonf_ < 0.003, Cohen’s d > 0.87).

Overall, the comparison between single tasks replicated the presence of a robust numerosity underestimation induced by symmetric cues and no effect of passive sensory load.

### The effect of divided attention

**Figure 3:**
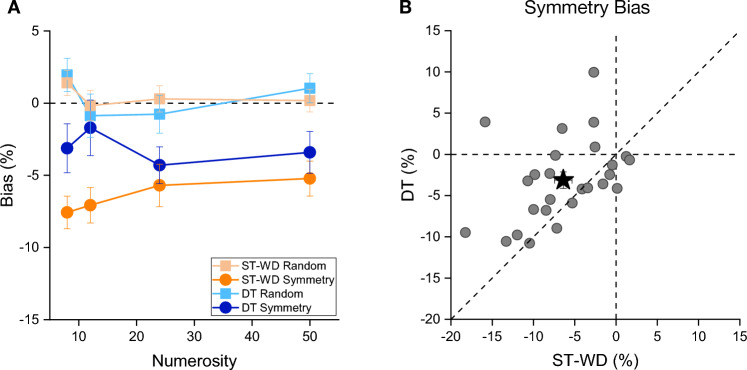
(**A**) Average Bias as a function of the standard numerosities, for the two conditions (symmetry and random) in single task with distractor (ST-WD) and dual-task (DT). (**B**) Averaged symmetry bias for each participant in the DT plotted against their ST-WD bias. The black star represents the average symmetry bias. Error bars represent ± 1 s.e.m.

Once replicated the symmetry-induced underestimation effect, we investigated whether this could be modulated by the availability of attentional resources. Given that the symmetry underestimation illusion was comparable between the conditions with and without the to-be-ignored distractor, for the subsequent analyses we used the single task with distractor to match the sensory load amongst the different experimental conditions. Figure 3A shows the underestimation bias in the single and dual-task conditions for both symmetric and random stimuli. From visual inspection it is evident that the curve reporting the bias found in the single-task condition is further apart compared to that reporting the result in the dual-task condition, suggesting a reduction of the effect of symmetry on perceived numerosity in the attention-deprived condition. The between participants average underestimation effect (across numerosity) induced by symmetry in the dual-task condition was ~ 50% smaller than that measured in the single task condition (single task: ~ 6%, dual-task: ~ 3%).

A RM ANOVA with numerosity (4 levels: 8, 12, 24, 50), condition (2 levels: symmetry and random) and task (2 levels: single and dual) as within-subjects factors confirmed a significant interaction between condition and task (F(1,25) = 7.25, p = 0.012, η^2^ = 0.017), indicating a different effect of attention across stimuli configurations. Neither the triple interaction (F(3,75) = 1.71, p = 0.17, η^2^ = 0.005), nor the interaction between numerosity and task (F(3,75) = 0.92, p = 0.44, η^2^ = 0.005), nor the interaction between numerosity and condition were statistically significant (F(3,75) = 1.45, p = 0.22, η^2^ = 0.008). Post-hoc t-tests showed that the underestimation bias was smaller for symmetric stimuli in the dual compared to single task condition (t(25) = 3.42, p_bonf_ = 0.008, Cohen’s d = 0.51) while no difference was observed for random arrays (t(25) = 0.09, p_bonf_ > 0.05, Cohen’s d = 0.014). Interestingly, the underestimation bias of symmetric arrays condition was reduced, but not cancelled, in the dual task, and it remained statistically different from the one measured with random arrays (t(25) = 3.19, p_bonf_ = 0.015, Cohen’s d = 0.54). These results suggest that reducing attentional resources with a DT diminished but did not annul the underestimation bias of symmetric stimuli. Figure 3B plots the individual data as bias in dual against single task conditions. Despite the large interindividual variability most of the datapoints falls above the equality line, indicating a reduced numerosity underestimation in the dual task compared to the single task, with attentional load (on average) halving the effect.

### Sensory precision

**Figure 4:**
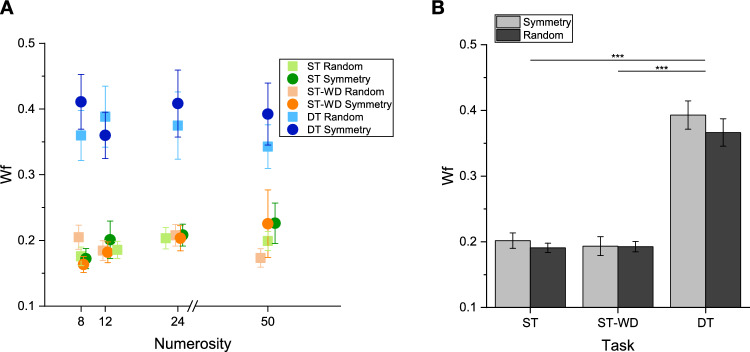
(**A**) Average Weber fraction as a function of the standard numerosities for the two conditions (symmetry and random) in single task (ST), single task with distractor (ST-WD) and dual-task (DT). (**B**) Weber fractions averaged across standard numerosities for each task and condition (symmetry: light grey bars; random: dark grey bars). ***p < 0.001. Error bars represent ± 1 s.e.m.

Once verified the effect of symmetry and attentional deprivation on the accuracy of numerical estimates, we analyzed their effect on sensory precision (Weber fraction; Wf). Figure 4 shows the average Wf as a function of the standard numerosities for the different experimental conditions. From visual inspection it is evident that precision deteriorated in the dual task condition, compared to the single task, across all the numerosity levels. On average (see Fig. 4B), Wfs in the dual task conditions were around 0.38 in the dual task and 0.20 in the single tasks (an increase of about 50%). To verify the differences across conditions, Wfs were entered in a RM ANOVA with numerosity (4 levels: 8, 12, 24, 50), condition (2 levels: symmetry and random) and task (3 levels: single task without distractor, single task with distractor, dual-task) as within-subjects factors. The main effect of task was statistically significant (F(2,50) = 62.42, p < 0.001, after Greenhouse–Geisser sphericity correction p < 0.001, η^2^ = 0.33) and post-hoc comparisons confirmed that Wfs were higher in dual task compared to both single task without (t(25) = 9.59, p_bonf_ =  < 0.001, Cohen’s d = 1.19) and with distractor (t(25) = 9.76, p_bonf_ =  < 0.001, Cohen’s d = 1.21), but no difference between the two single tasks (ST vs ST-WD: t(25) = 0.18, p_bonf_ > 0.05). Importantly, Wfs did not differ between symmetry and random conditions (no main effect of condition: F(1,25) = 2.42, p = 0.13, η^2^ = 0.002), suggesting that the underestimation effects were not due to differences in the difficulty between the two conditions. Finally, the main effect of numerosity was not significant (F(3,75) = 0.78, p = 0.51, after Greenhouse–Geisser sphericity correction p = 0.49, η^2^ = 0.003) as Wfs remained constant across numerosities (Fig. 4A). None of the interactions were statistically significant (F < 1.09, p > 0.36, η^2^ < 0.005).

### Color–orientation conjunction task performance

**Figure 5:**
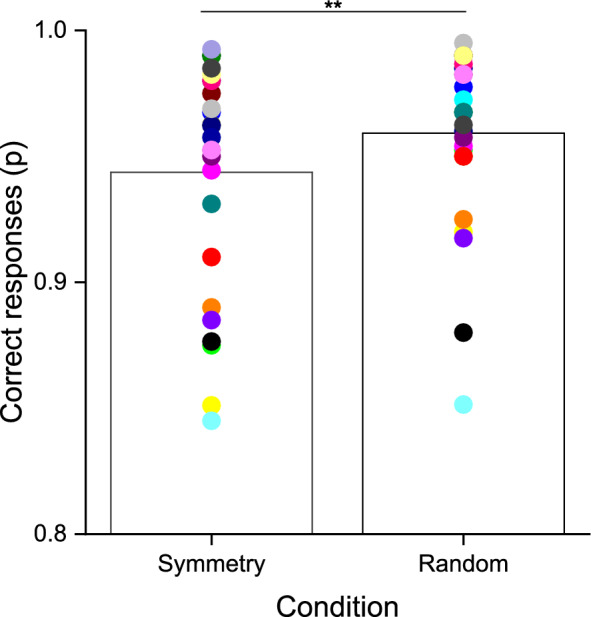
Color–orientation conjunction task performance. Proportion of correct responses for symmetry and random conditions respectively. Dots represent individual participants (color coded).

As a final analysis, we evaluated the performance (correct responses) in the color–orientation conjunction task, when it was carried out in conjunction with the numerical comparison task (Fig. 5). The between participants’ average performance was high both when the color–orientation conjunction task was performed during the numerical comparison of random (mean: 0.96; std: 0.03) and symmetrical stimuli (mean: 0.94; std: 0.04). To statistically compare the two conditions, we performed a Wilcoxon signed-rank test on correct responses. The results indicated a higher performance in the distractor task when it was performed in conjunction with numerical comparison of random, compared to symmetrical stimuli (W = 59; p = 0.006), potentially suggesting a bidirectional interference between the numerical and non-numerical stimuli in the symmetry condition.

## Discussion

The current study investigated the origin of the symmetry-induced numerosity underestimation effect by evaluating its reliance on visual attention. Participants compared the numerosity of symmetric and random dot patterns while no distractor were presented onscreen (single task, ST); while visual distractor were presented but had to be ignored (single task with ignored distractor, ST-WD); and while performing a concurrent color-orientation task on the visual distractor (dual task, DT). We replicated previous studies by showing that, when visual resources were fully available, symmetric patterns were numerically underestimated compared to random patterns^22,49^. We further observed that this effect persisted even when a visual distractor was presented onscreen but had to be ignored, suggesting that merely increasing the sensory load was not sufficient to decrease the strength of the illusion. Interestingly, despite the precision in numerosity discrimination of symmetric compared to random stimuli remained unaltered when visual attention was diverted by a color–orientation conjunction task, the numerosity underestimation of symmetric stimuli halved, consistent with the hypothesis that the symmetry-induced underestimation relies on attentional-dependent grouping mechanisms.

Symmetry is a crucial Gestalt principle for object’s identification and segregation^50^, which is necessary for numerosity perception^53^. Previous studies have shown that other Gestalt cues can affect numerosity perception. Specifically, grouping cues such as proximity, common region, and connectedness lead to systematic underestimation of perceived numerosity, with underestimation effects ranging from around 18% for common region^24,30^, to 20% for proximity^24^ and to 20–30% for connectedness, depending on the number of connected elements^24–30,34^. The underestimation effect triggered by symmetry is much weaker, only around 8–10% when attentional resources are fully available^22,49^. This weaker effect is probably related to the fact that symmetry detection might rely on long range interactions and point-by-point matching across the visual field. Yet, irrespective of the specific mechanisms supporting symmetry detection, it is well documented that symmetry is easier and faster to detect when elements are closer to the vertical symmetry axis, especially if they are in the center of the screen, as in the case of the current experiment^54^. Therefore, the impact of symmetry on numerosity perception might be stronger for dots closer to the vertical axis (i.e., the axis of reflection) and gradually decrease for a more peripheral pairs of dots. This might result in an overall weaker underestimation effect compared to the one elicited by other spatial-grouping cues that might rely on more local grouping mechanisms which integrate regions of homogeneous texture (generally belonging to the same object)^55^ over an estimated window of approximately 4°^56^.

Interestingly, numerosity underestimation seems to be elicited only by spatial-grouping cues (connectedness, proximity, common region) and not by feature-similarity grouping cues, such as color^24,30^, shape or even the conjunction of both^24^, at least when items are simultaneously presented^57^. Yu et al.^24^ interpreted this result as evidence in support of feature-similarity grouping being a serial process carried out by feature-based attention, as it was not able to interfere with a simultaneous and relatively attention-free process as numerosity perception^34,58,59^. On the contrary, the numerosity underestimation driven by the spatial-grouping cues was taken as evidence of interference between numerosity perception and Gestaltic principles that identify groups simultaneously and pre-attentively. Yu et al.^24^ did not test the impact of symmetry on numerosity underestimation, and the current results do not allow us to list symmetry amongst any of the two grouping categories proposed by Yu et al. (either spatial-grouping or feature similarity grouping). Indeed, on the one hand, symmetry can be considered as providing spatial grouping cues, because it relies on the organization of items in space rather than on feature similarity, however, on the other hand, we found a clear attentional modulation of the numerosity underestimation, suggesting that symmetric groups might not be extracted pre-attentively and in parallel across the visual field.

The current results fit better with the incremental grouping theory^60^ which proposed that there are two forms of perceptual grouping: base grouping and incremental grouping. Base grouping is fast, occurs in parallel across the visual image and relies on the feedforward activation of neurons that are tuned to some features or feature conjunctions (for example color, orientation or the conjunction of color and orientation). However, when there are no neurons directly tuned to a specific feature or feature conjunctions, such as in the case of symmetry or of the conjunction between numerosity and symmetry, a second grouping process, called incremental grouping, is activated. Incremental grouping is time-consuming, capacity-limited, and attention-dependent. During the incremental grouping process, neurons coding for features that are grouped together would enhance their responses through recurrent connections to, for example, facilitate the co-selection of contour elements which identify complex shapes. According to this view, incremental grouping would rely on the spread of attention across elements that belong to the same perceptual object. We suggest that the symmetry-induced underestimation of numerosity might be elicited by attention-dependent mechanisms such as the ones mediating incremental grouping. Performing the concurrent color–orientation conjunction task reduced the attentional resources available and this might have interfered with participants’ ability to detect groups defined by symmetry, thus reducing the underestimation effect.

In the present study, we aimed at investigating the origin of the symmetry-induced numerosity underestimation effect and its dependence on attentional resources. Although this study was not designed to investigate the role of attention in symmetry perception, our results indirectly contribute to the debate in the literature about whether attention is needed for symmetry perception. Classical studies found that, in figure-ground segregation tasks, symmetric figures were detected faster and easier than asymmetric ones, even after very brief presentation of stimuli, suggesting that symmetry perception might occur pre-attentively^45,48,50,61–64^. However, these results are difficult to reconcile with studies showing that symmetry detection is facilitated by long presentation time or dynamic flicker^50,64–66^. Moreover, a recent study using multi-color patterns showed that symmetry detection improved when participants’ attention was directed to the color of the symmetric patterns, suggesting that symmetry benefits from feature-based attention (in this case attention to color)^67^. Taken together, these studies point at the existence of additional slow and sequential mechanisms for symmetry detection which process the spatial distribution of attentionally selected features^64,68^.

Overall, while there is clear evidence for symmetry, as well as for other grouping principles to organize scene perception pre-attentively^37,39,69–73^, attention might nevertheless modulate symmetry perception to some extent. In this view, the current results provided an example of the role of attention in activating symmetry-based grouping mechanisms that, in turn, seem to trigger a significant numerosity underestimation.

It is important to note, however, that the underestimation effect was only halved, and not annulled, by the dual-task. This might suggest that symmetry could still be detected to some extent under attentional load, at least with the current concurrent task. This was observed also in a previous study that used the very same dual-task which, again, only halved the numerosity underestimation elicited by element connectedness^34^. Considering that performance to the color–orientation conjunction task was very high, future studies should use a more challenging task.

By analyzing the performance to the color–orientation conjunction task, we found that accuracy was lower when it was concurrent with numerosity discrimination of symmetrical arrays compared to the random condition. This result might be explained by the presence of symmetric cues also in the color–orientation conjunction task—targets and non-targets were defined by specific (symmetric) arrangements of colored squares around the fixation point. Importantly, even if the color–orientation conjunction task might have been slightly more difficult for the symmetric compared to the random condition, numerosity precision was comparable across conditions, making unlikely that difficulty could account for the reduced underestimation effect under dual-task.

Future studies should investigate at what level of the visual processing the interaction between numerosity and symmetry occurs. So far, only the neural substrate of another grouping-induced numerosity underestimation illusion, the connectedness illusion, has been reported^30,74^. In a combined fMRI and EEG study, Fornaciai and Park^74^ found that the effect of connectedness arose from the EEG signal 150 ms after the stimulus onset and could be decoded from the pattern of activity read out from area V3, suggesting that the interaction between grouping mechanisms and numerosity perception occurs at middle-level of visual processing. Up to date, the interaction between the number of items in a display and symmetry has been investigated only with respect to symmetry perception. fMRI studies found that BOLD activity scaled with the proportion of symmetric vs random dot patterns as well as with the number of symmetry axes^75,76^ in several extra-striate areas, including V3A, V4, V7, the lateral occipital complex (LOC)^75,76^ and more ventral and temporal regions (i.e., VO1, VO2, TO1 and TO2)^76^. Moreover, response to symmetry in these regions was modulated by attention: compared to passive viewing, diverting attention from the symmetric stimuli by means of a color-detection task reduced the symmetry-related response^75^, whereas directing attention to symmetry by means of symmetry-detection task increased the symmetry-related responses^76^. These results are consistent with ERPs studies showing that the proportion of symmetric vs random dot patterns elicited a late and sustained negative component (i.e., sustained posterior negativity or SPN) thought to arise from intermediate stage of visual processing^77^ and that this component was modulated by feature-based attention^78^. We therefore speculate that also the symmetry-induced underestimation effect might arise at middle level of visual processing, similarly to the connectedness illusion.

In conclusion, the present study suggests that symmetry affects numerosity perception only after an attention-dependent mechanism has integrated multiple parts of the visual field into perceptual objects. The current study highlights a complex relationship between numerosity, symmetry and attention, three fundamental mechanisms to reconstruct an accurate and coherent representation of the external world.

## Data Availability

Data for the main findings are available at: 10.5281/zenodo.8197028 (accessed on 30th July 2023).
